# Proximal Arteriovenous Fistulas in Hemodialysis Patients: Advantages and Disadvantages

**DOI:** 10.7759/cureus.11657

**Published:** 2020-11-23

**Authors:** Cengiz Güven, Öznur Uludağ

**Affiliations:** 1 Cardiac/Thoracic/Vascular Surgery, Adıyaman University Faculty of Medicine, Adıyaman, TUR; 2 Anesthesiology and Reanimation, Adıyaman University Faculty of Medicine, Adıyaman, TUR

**Keywords:** hemodialysis, complication, brachiocephalic fistula, basilic vein transposition

## Abstract

Introduction: The main objective of the present study is to investigate the advantages and disadvantages of proximal arteriovenous native fistulas. Hemodialysis is indispensable for patients with end-stage renal disease. For this purpose, arteriovenous fistulas (AVFs) are used. Among the native fistulas, distal radiocephalic AVF is the most preferred. However, brachiocephalic AVF (BCAVF) and brachiobasilic AVF with basilic vein transposition (basilic vein transposition arteriovenous fistula [BVTAVF]) can be used for a long time in dialysis patients whose distal vascular bed is depleted.

Methods: This is a retrospective study of 117 AVFs (BCAVF and BVTAVF), in patients with end-stage chronic renal disease, that were opened with a surgical technique (2012-2018). The postoperative two-year patency rates, AVF locations, complications, and the advantages and disadvantages of these fistulas are reviewed and recorded in the light of the literature.

Results: The mean age of the patients (52 men and 65 women) was 60.6 ± 13.6 years. The percentages of primary patency rates at 3, 6, 9, 12, and 24 months were 96.6%, 93.1%, 92%, 87.4%, and 82.8% in BCAVF patients, and 96.7%, 93.3%, 90%, 86.7%, and 80% in BVTAVF patients, respectively. The percentages of secondary patency rates at 6, 12, and 24 months were 100%, 93.3%, and 86.7% in BCAVF patients, and 100%, 100% and 87.7% in BVTAVF patients, respectively. Fistula thrombosis was seen as the most common complication. The early complication was bleeding/hematoma. As late complications, we encountered steal syndrome, ischemic pain in the relevant extremity, pseudoaneurysm, and high-output heart failure.

Conclusion: Proximal AVFs are preferable fistulas with early maturation and high primary patency rates. We believe that relatively high complications can be avoided by opening fistulas with an appropriate surgical technique.

## Introduction

Hemodialysis patients with central venous catheters have larger mortality due to infections. But the hemodialysis patients with native arteriovenous fistulas (AVFs) have a long period of patency and less complications. Therefore, for patients whose life is dependent on hemodialysis, AVFs are the most preferred renal replacement methods. A distal radiocephalic AVF is the frequently preferred vascular access technique among fistulas [1]. Proximal fistulas opened using the brachial artery as the artery and the cephalic and basilic vein as the vein are alternative vascular access routes in patients with an insufficient or depleted distal vascular bed [2]. Brachiocephalic AVF (BCAVF) and brachiobasilic AVF with basilic vein transposition (basilic vein transposition arteriovenous fistula [BVTAVF]) can be used for a long time with their excellent potency ratio [3]. These fistulas have some disadvantages and advantages. Beside the advantages such as early maturation and earlier dialysis start, BCAVF has disadvantages such as steal syndrome, venous hypertension, and edema in the arm, and BVTAVF has disadvantages such as bleeding, hematoma, aneurysm formation, and steal syndrome [4-6].

The main objective of this study is to examine patients with proximal AVFs and share AVFs' advantages and disadvantages in the light of the literature.

## Materials and methods

This study was carried out in the Adıyaman University Medical Faculty Cardiovascular Surgery Clinic, Adıyaman, Turkey. A total of 117 patients with proximal AVF opening were admitted. All patients with autologous proximal AVF and over the age of 18 between June 2012 and December 2018 were included in the study. Patients with distal AVFs and AVFs opened with synthetic graft were not included in the study. This was a retrospective cohort study and the data collected were used for study purposes only. This study was approved by the Institutional Review Board of the Adıyaman University Medical Faculty, where the study was conducted (approval no. 2020/1-5). All surgical procedures are recorded in our database. The patients who had proximal AVF operated in our clinic between June 2012 and December 2018 were evaluated in terms of fistula location, dialysis initiation times, complications, advantages and disadvantages, and primary and secondary patency rates. The access was considered mature and successful with a flow rate of at least 350 mL/min at the control with colored venous Doppler ultrasonography and it was recorded as the first dialysis time in the postoperative one- and two-month follow-ups of the patients.

Statistical analyses

Statistical analysis to determine patency rates was performed by Kaplan-Meier method using the GraphPad Prism 8.0.2 (GraphPad Software, Inc., CA; retrieved from http://www.graphpad.com/scientific-software/prism/). An independent two-sample t-test was used to compare the first cannulation time. This result was reported as mean±SD. Categorical variables were compared using the chi-square homogeneity test and chi-square independence test. Data were expressed as counts and percentages. A p value <0.05 was considered statistically significant.

Surgical technique

**Figure 1:**
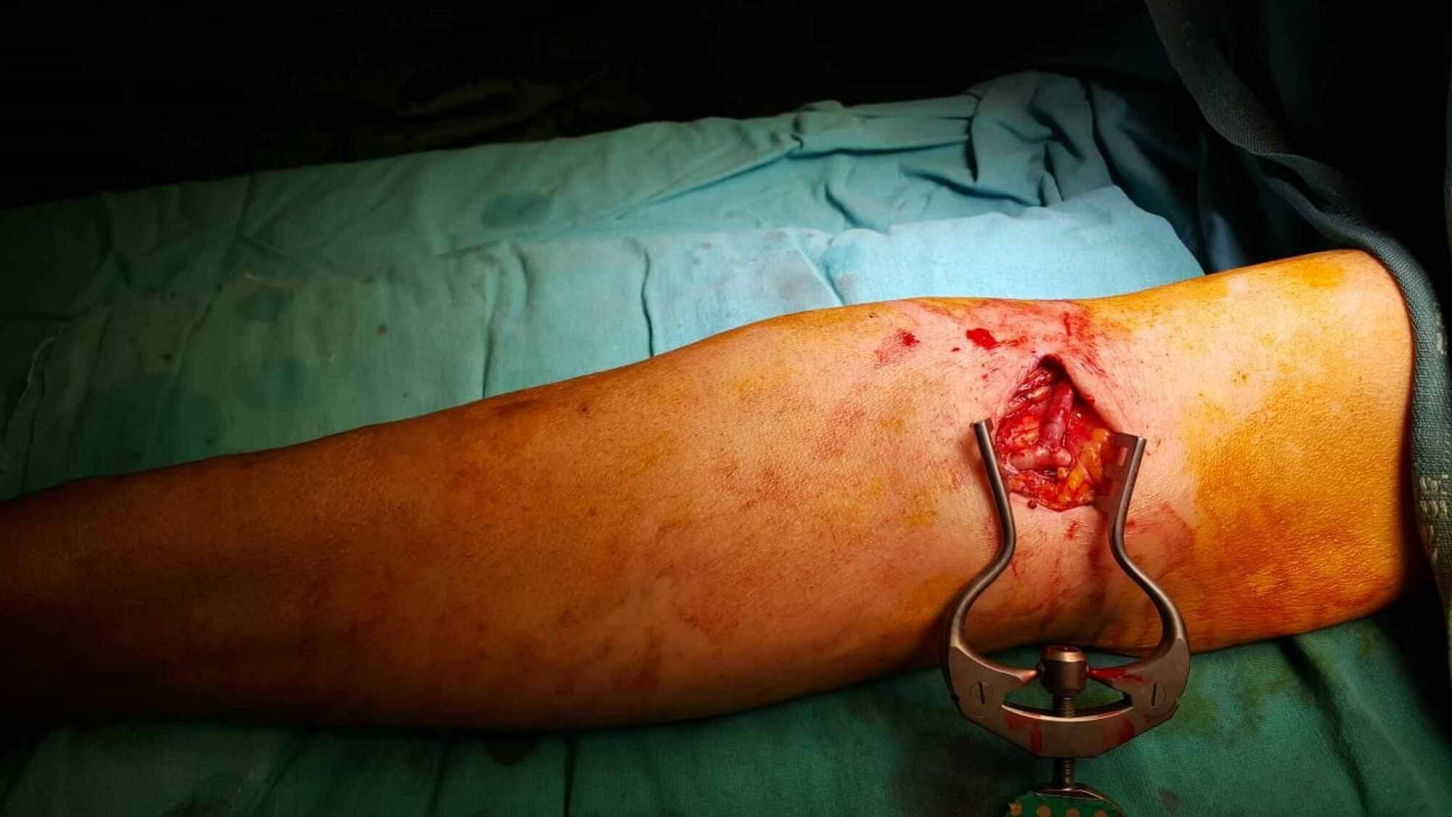
Brachiocephalic arteriovenous fistula

BCAVF patients were operated under local anesthesia, while BVTAVF patients were operated with an axillary block. Blood pressure, pulse, and pulse oximetry monitoring was performed in all patients. In BCAVF, following appropriate staining and covering, an appropriate incision (approximately 3.5-4 cm) was made in the antecubital region of the relevant extremity. The cephalic vein and brachial artery were found and turned. After the cephalic vein was released, its distal part was tied and cut. Lumen was washed with heparinized fluid. Systemic 5000 IU heparin was administered to the brachial artery. Vascular clamps were placed five minutes later. Approximately a 3.5-4 mm arteriotomy was performed. The cephalic vein was anastomosed to the brachial artery using an end-to-side anastomosis technique (Figure 1).

Then, the subcutaneous tissue and skin were closed with appropriate sutures.

**Figure 2:**
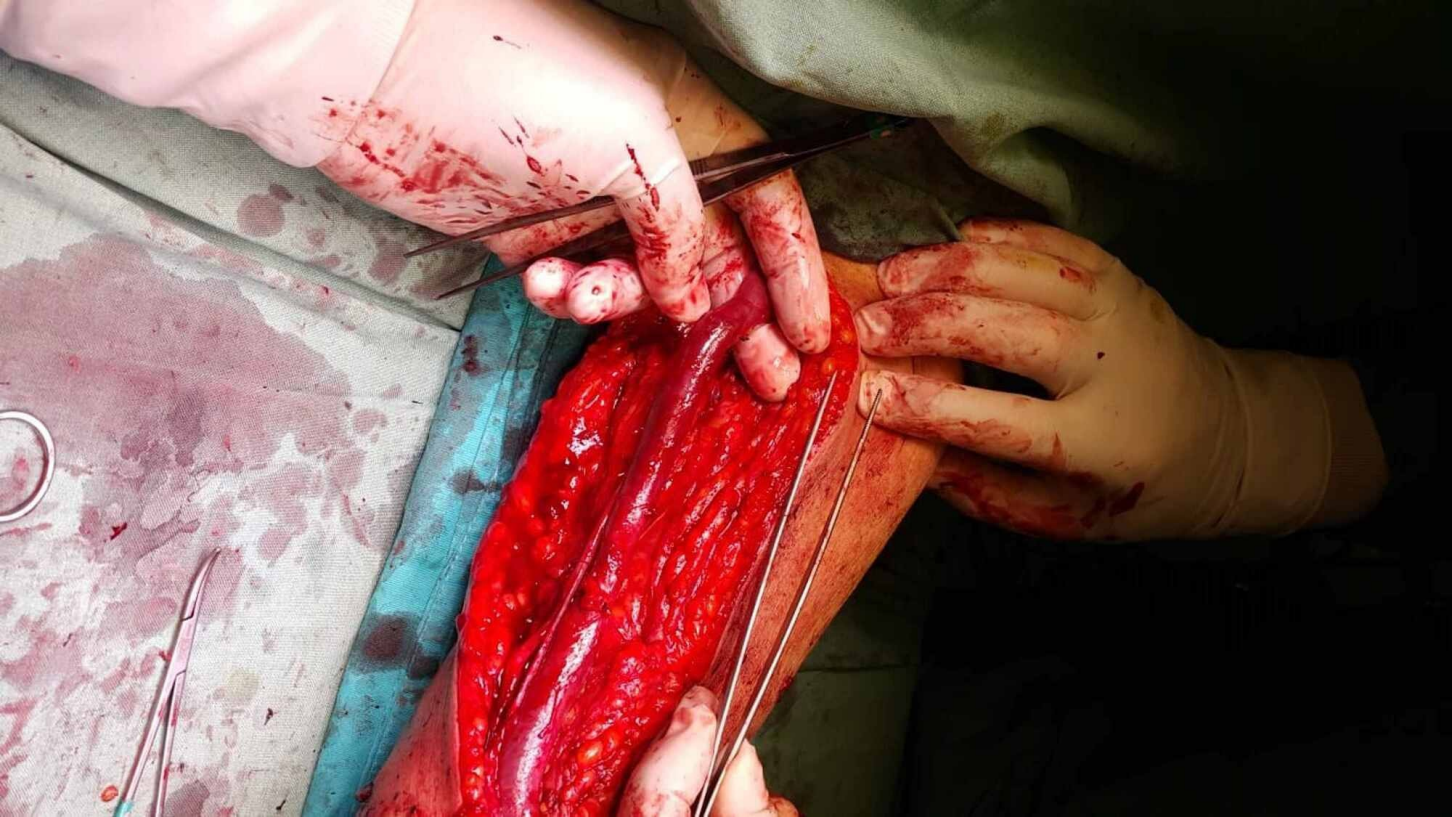
Basilic vein transposition arteriovenous fistula

Following appropriate staining and covering, an appropriate skin incision was made from the antecubital area of the relevant extremity in BVTAVF. The basilic vein was found, turned, and explored in the axillary region until it was poured into the axillary vein. The lateral branches were tied and released (Figure 2).

Lumen was washed with heparinized fluid. The brachial artery was found and turned. The basilic vein was passed through a subcutaneous tunnel in the lateral region. After 5000 IU systemic heparin was given, vascular clamps were placed in the brachial artery. A 3.5-4 mm arteriotomy was performed on the brachial artery. The basilic vein passed through a tunnel under the skin was anastomosed to the brachial artery using an end-to-side anastomosis technique. After the procedure, a closed vacuum drain was placed in the lodge and subcutaneous and skin was closed with appropriate sutures.

In both surgical techniques, arteriotomy for anastomosis was performed at a level of <5 mm as possible, thus minimizing distal extremity ischemia.

## Results

A total of 117 patients underwent proximal AVF in six years. There were 65 females and 52 males in our study. The patients showed a homogeneous distribution in terms of gender (p>0.05). The mean age was 60.6±13.6 years. Operative and demographic data of the patients are summarized in Table 1.

**Table 1:** Operative and demographic features BCAVF: brachiocephalic arteriovenous fistula; BVTAVF: basilic vein transposition arteriovenous fistula; BMI: body mass index ^a^A chi-square homogeneity test was used. ^b^An^ ^independent two-sample t-test was used.

		Mean (SD), n (%)	p value
Age (year)	60.6±13.6		
Gender^a^			
Male	52 (44.4)		0.229
Female	65 (55.6)		
Target arm^a^			
Left forearm	100 (85.5)		<0.0001
Right forearm	17 (14.5)		
AVF position^a^			
Left BCAVF	73 (62.4)		
Right BCAVF	14 (12)		<0.0001
Left BVTAVF	27 (23.1)		
Righ BVTAVF	3 (2.5)		
Concomitant diseases			
Diabetes mellitus	47 (40.2)		
Hypertension	74 (63.2)		
Coronary artery disease	26 (22.2)		
Atherosclerosis	37 (31.6)		
Hyperlipidemia	29 (24.8)		
Morbid obesity (BMI ≥ 40)	24 (20.5)		
Peripheral arterial disease	39 (33.3)		
First cannulation time (day)^b^			
BCAVF	41.3±9.7		0.050
BVTAVF	37.9±9.2		

BCAVF was opened in 87 patients and BVTAVF was opened in 30 patients. There was no homogeneous distribution in terms of the target arm (p<0.001). It was determined that the left forearm (85.5%) was significantly more preferred than right forearm (14.5%) (p<0.001). There was no homogeneous distribution in terms of AVF position (p<0.001). Left BCAVF (62.4%) and left BVTAVF (23.10%) were seen significantly higher than other graft positions (p<0.001). The time to start dialysis was determined as 41.3±9.7 days in BCAVF and 37.7±9.2 days in BVTAVF (p=0.05). It was found that 109 (93.2%) patients had previously opened multiple AVF from the distal regions and thrombosed over time. In the other 8 (6.8%) patients, it was found that the distal vascular bed was insufficient and the proximal AVF was opened because the vascular structures were extremely thin. The most common causes of renal failure were hypertension and diabetes mellitus (Table 2).

**Table 2:** Cause of end-stage renal disease

Cause	n (%)
Primary glomerulonephritis	3 (2.6)
Diabetic nephropathy	23 (19.7)
Polycystic renal disease	5 (4.3)
Obstructive stone disease	2 (1.7)
Hypertension	39 (33.3)
Pyelonephritis	3 (2.6)
Autoimmune disease	2 (1.7)
Congenital renal disease	4 (3.4)
Renovascular disease	12 (10.2)
Various	11 (9.4)
Unknown	13 (11.1)

The most common complications in the first three months were bleeding and hematoma in both BCAVF (12 patients) and BVTAVF (4 patients). It was found that all of these patients recovered without intervention requirement. During the follow-up period, the total infection rate was approximately 5%. The fistula thrombosis developed in 21 patients out of 117 patients, with 15 patients with BCAVF and 6 patients with BVTAVF. Hand ischemia due to steal syndrome developed in six patients (6.9%) with BCAVF and two patients (6.7%) with BVTAVF (Table 3).

**Table 3:** Complications at follow-up months

Months	Complications	BCAVF, n=87	BVTAVF, n=30	Total, n (%)	p value
3	Thrombosis	3	1	4 (3.4)	0.976
	Bleeding/hematoma	12	4	16 (13.7)	0.950
	Hand ischemia/steal	2	0	2 (1.7)	0.402
	Infection	4	2	6 (5)	0.658
	Venous hypertension	0	1	1 (0.9)	0.087
6	Thrombosis	3	1	4 (3.4)	0.976
	Pseudoaneurysm	2	1	3 (2.7)	0.757
	Hand ischemia/steal	3	0	3 (2.7)	0.303
9	Thrombosis	1	1	2 (1.7)	0.426
	Pseudoaneurysm	2	0	2 (1.7)	0.402
	High output heart failure	1	0	1 (0.9)	0.555
	Venous hypertension	5	3	8 (6.8)	0.426
	Venous aneurysm	1	2	3 (2.7)	0.099
12	Thrombosis	4	1	5 (4.3)	0.792
	Hand ischemia/steal	1	2	3 (2.7)	0.099
	Venous hypertension	1	1	2 (1.7)	0.426
	Venous aneurysm	7	2	9 (7.7)	0.807
24	Thrombosis	4	2	6 (5.1)	0.658
	Pseudoaneurysm	0	2	2 (1.7)	0.015
	Venous aneurysm	2	2	4 (3.4)	0.256

**Figure 3:**
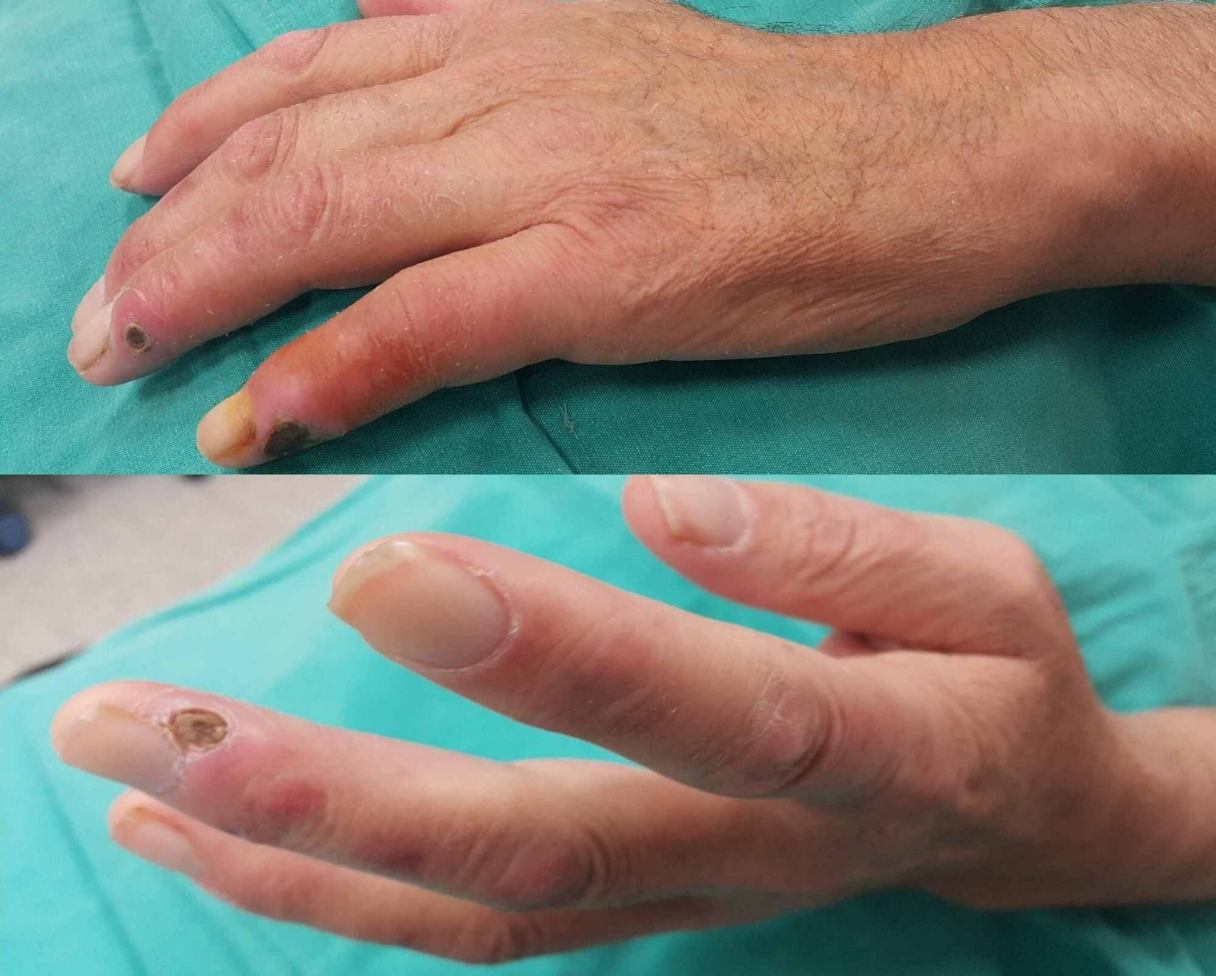
Hand ischemia

It was observed that banding was applied to three patients with ischemia, and two patients were relieved with medical treatment (Figure 3).

**Figure 4:**
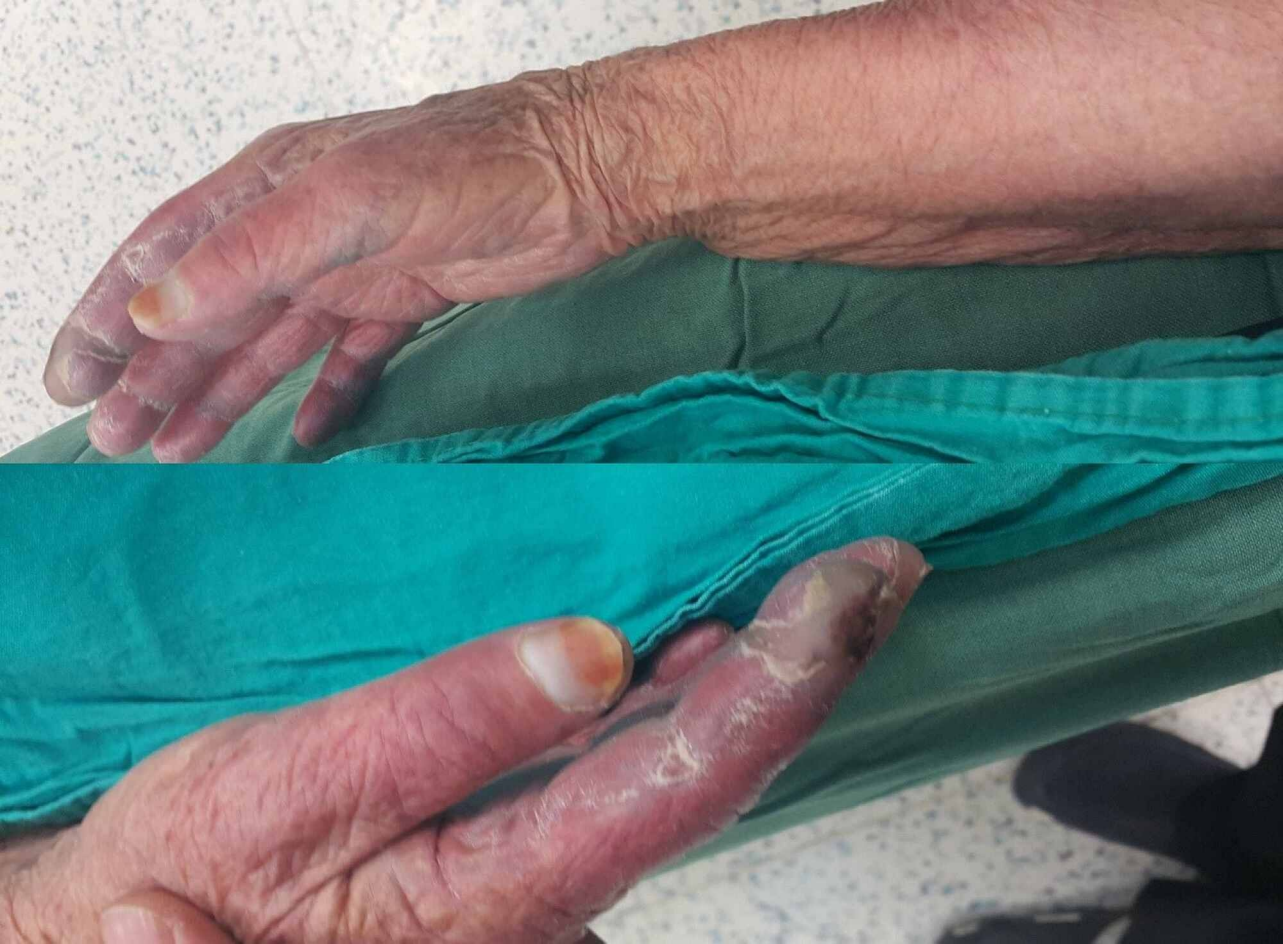
Steal syndrome

It was found that the fistula of three patients developed steal syndrome and they had severe pain, edema and ischemic symptoms (Figure 4).

**Figure 5:**
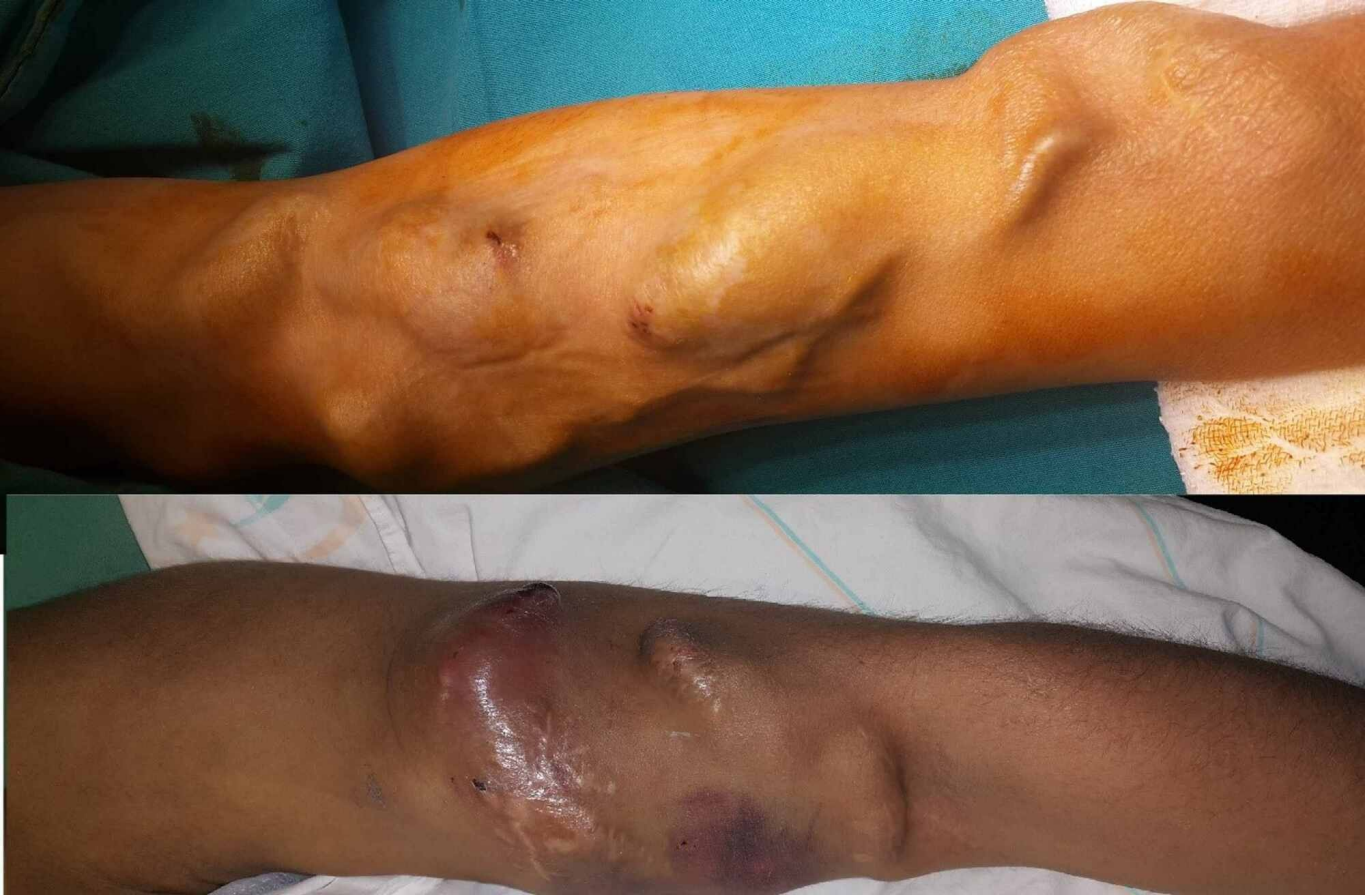
Venous aneurism

Venous aneurysm developed in 16 patients during the follow-up period (Figure 5).

**Figure 6:**
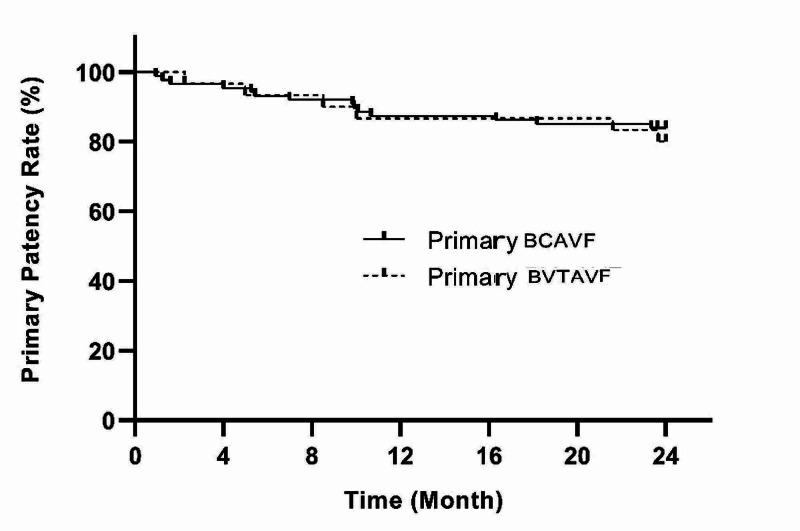
Primary patency rate BCAVF: brachiocephalic arteriovenous fistula; BVTAVF: basilic vein transposition arteriovenous fistula

In the echocardiography performed on a patient who came to the polyclinic with shortness of breath, heart failure due to high output was found in the ninth month. As a matter of fact, the fistula flow rate in the color Doppler ultrasonography performed on this patient was 2300 mL/min. The fistula of this patient had to be closed. There was no significant difference in the complications at follow-up months except for pseudoaneurysm (p>0.05). Pseudoaneurysm was seen more significantly in BVTAVF (p<0.05) (Table 3). The percentages of primary patency rates at 3, 6, 9, 12, and 24 months were 96.6%, 93.1%, 92%, 87.4%, and 82.8% in BCAVF patients, and 96.7%, 93.3%, 90%, 86.7%, and 80% in BVTAVF patients, respectively (Figure 6).

**Figure 7:**
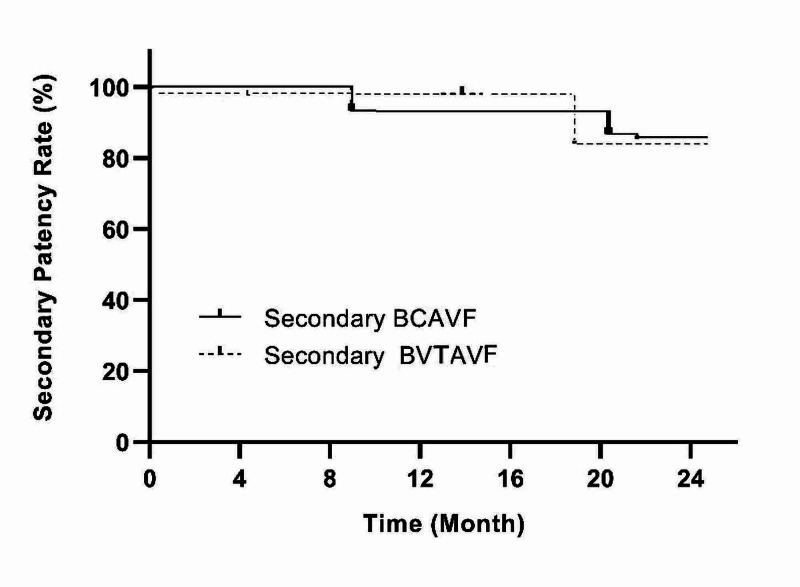
Secondary patency rate BCAVF: brachiocephalic arteriovenous fistula; BVTAVF: basilic vein transposition arteriovenous fistula

The percentages of secondary patency rates at 6, 12, and 24 months were 100%, 93.3%, and 86.7% in BCAVF patients, and 100%, 100%, and 87.7% in BVTAVF patients, respectively (Figure 7).

## Discussion

Hemodialysis patients need more than one vascular access procedure throughout their lives. Autogenous AVFs are the most preferred vascular access for this purpose [7]. They are preferred more frequently than nonautogenous grafts and central catheters due to their high long-term patency rates and lower risk of thrombosis and infection [8,9]. According to the National Kidney Foundation - Dialysis Outcomes Quality Initiative (NKF-DOQI) and the Society for Vascular Surgery's 2008 Clinical Practice Guidelines, the order of preference is distal radiocephalic, elbow BCAVF, and BVTAVF [10,11]. In 109 of 117 patients in our study, it was observed that proximal BCAVF and BVTAVF were switched over to after distal AVFs were opened and thrombosed over time. In the other eight patients in the study, proximal AVF was preferred because the distal vascular bed was not good. In a study reported by Başer et al., early and late success rates of AVF in the proximal were 90.3% and 84.9%, respectively, while they were 76.9% and 69.2% in the distal. In this study, 31 (23.5%) complications were observed in proximal fistulas. Early thrombosis was found in 18 (13.6%), late thrombosis in 8 (6.1%), bleeding in 2 (1.5%), infection in 2 (1.5%) and pseudoaneurysm in 1 (0.8%) [12]. In another study by Wen et al., primary patency rates were reported to be 83.3% and 75%, respectively, during the three- and six-month follow-up periods for BCAVFs opened proximally. In this study, the early thrombosis rate was 18.8% [13]. In a meta-analysis by Sheta et al., primary patency rates for BVTAVF were found to be approximately 85% [14]. In the literature review, the primary patency rate of primary fistulas was found to be 75% to 90.3% [12-14]. In our study, the percentages of primary patency rates at 3, 6, 9, 12, and 24 months were 96.6%, 93.1%, 92%, 87.4%, and 82.8% in BCAVF patients, and 96.7%, 93.3%, 90%, 86.7%, and 80% in BVTAVF patients, respectively. The percentages of secondary patency rates at 6, 12, and 24 months were 100%, 93.3%, and 86.7% in BCAVF patients, and 100%, 100%, and 87.7% in BVTAVF patients, respectively. These rates are higher than those found in the literature. We attribute that all fistulas in the study were performed by a single surgical team. To our knowledge, our health center is the only center performing fistula surgery in the region and our surgical team is the only team with experience in this field, increasing the success of fistula surgery. Early thrombosis (within the first three months) was observed in 3.4% of our patients, and late thrombosis (between three months and two years) was observed in 14.6% of our patients. In the literature review, high complications in BVTAVF are mentioned and this is connected to long incisions used for basilic vein exploration. Complication rates are between 47% and 71% [15]. In our study, the general complication rates in the first three months were determined as 24% in BCAVF and 26% in BVTAVF. It was observed that the most common complications were bleeding and hematoma. None of these patients required intervention. The most important and only technical difficulty in BVTAVF is to perform surgery for obese patients and access fistula for hemodialysis in patients with thick subcutaneous adipose tissue [16]. Edema or venous hypertension is another disadvantage of proximal fistulas. This is mostly due to extensive incisions and cutting of dense collateral veins or thrombosis of these veins over time. This may cause edema by reduction in venous return in the extremity [17]. Edema makes fistula access difficult during dialysis. It can also cause subcutaneous bleeding and hematomas due to multiple attempts. In a study by Veeramani et al., the incidence of edema was found to be 14.2% [18]. In our study, arm edema (venous hypertension) occurred in 11 patients out of 117 patients during the follow-up period. There were 6 (5%) patients in the BCAVF and 5 (16.7%) patients in BVTAVF group. No additional intervention was made to any of these patients. It was followed by effective elevation. Another complication in fistula surgery is arterial steal syndrome. This is especially seen in patients with diabetic and atherosclerotic vascular disease and proximal fistulas [19,20]. The rate of steal syndrome requiring intervention in the literature is approximately 2.9% to 3.9%. In the treatment of this condition, which has the risk of loss of extremity, it requires banding or complete ligation of the fistula [21]. In our series, this rate was found to be 6.9% in BCAVF patients and 6.7% in BVTAVF patients. Because three of these patients had severe pain and limb ischemia, the fistula had to be closed. It was found that two of the other five patients were relieved by medical treatment, and three of them had their fistula narrowed by taping method. These taped patients continued to use their fistulas without any problems. Two-year secondary patency rates were >85%. Aneurysms, which are one of the late fistula complications in dialysis patients, are generally true aneurysms and their etiology is not clear. However, repeated needles or connective tissue disorder in the same area is to be blamed. Their incidence varies between 5% and 60% [22-25]. In our study, after the sixth month, true venous aneurysms were detected at 11.5% in BCAVF patients and 20% in BVTAVF patients. These rates were similar to the literature. Our pseudoaneurysm rate was 6% with seven patients at the two-year follow-up. In such aneurysms, it occurs with the weakening of the vascular wall as a result of repeated punctures performed in the same area during dialysis [26]. High-output heart failure is a rare but serious complication described in the literature. There is high evidence that a fistula with a high output, such as worsening or inducing left ventricular hypertrophy, can contribute to the high cardiovascular risk factor present in patients with renal insufficiency [27,28].

This complication, which was observed in 0.9% with only one patient in our study, was treated with fistula closure. After the fistula is formed, a maturation period is expected for fistula cannulation for an average of three to six months. Meanwhile, the patient starts dialysis with a central venous catheter. This brings additional complications [29]. This period was 41.3±9.7 days in BCAVF patients and 37.9±9.2 days in BVTAVF patients in our study group. This seemed to be the most important advantage of proximal fistulas.

## Conclusions

Proximal arteriovenous fistulas appear to be advantageous with high primary patency rates, secondary patency rates and early dialysis times. We believe that aneurysm formation and steal syndrome, which are relatively high in proximal compared to distal arteriovenous fistulas, can be prevented by appropriate surgical techniques and punctures in different regions during dialysis. Proximal arteriovenous fistulas are an appropriate vascular access route in patients whose distal vascular bed is depleted or unsuitable.
